# Role of type 1 and type 3 fimbriae in *Klebsiella pneumoniae *biofilm formation

**DOI:** 10.1186/1471-2180-10-179

**Published:** 2010-06-23

**Authors:** Casper Schroll, Kim B Barken, Karen A Krogfelt, Carsten Struve

**Affiliations:** 1 Department of Microbiological Surveillance and Research, Statens Serum Institut, 2300 Copenhagen S, Denmark; 2 Centre for Biomedical Microbiology, Technical University of Denmark, 2800 Lyngby, Denmark

## Abstract

**Background:**

*Klebsiella pneumoniae *is an important gram-negative opportunistic pathogen causing primarily urinary tract infections, respiratory infections, and bacteraemia. The ability of bacteria to form biofilms on medical devices, e.g. catheters, has a major role in development of many nosocomial infections. Most clinical *K. pneumoniae *isolates express two types of fimbrial adhesins, type 1 fimbriae and type 3 fimbriae. In this study, we characterized the role of type 1 and type 3 fimbriae in *K. pneumoniae *biofilm formation.

**Results:**

Isogenic fimbriae mutants of the clinical *K. pneumoniae *isolate C3091 were constructed, and their ability to form biofilm was investigated in a flow cell system by confocal scanning laser microscopy. The wild type strain was found to form characteristic biofilm and development of *K. pneumoniae *biofilm occurred primarily by clonal growth, not by recruitment of planktonic cells. Type 1 fimbriae did not influence biofilm formation and the expression of type 1 fimbriae was found to be down-regulated in biofilm forming cells. In contrast, expression of type 3 fimbriae was found to strongly promote biofilm formation.

**Conclusion:**

By use of well defined isogenic mutants we found that type 3 fimbriae, but not type 1 fimbriae, strongly promote biofilm formation in *K. pneumoniae *C3091. As the vast majority of clinical *K. pneumoniae *isolates express type 3 fimbriae, this fimbrial adhesin may play a significant role in development of catheter associated *K. pneumoniae *infections.

## Background

*Klebsiella pneumoniae *is an important gram-negative opportunistic pathogen causing primarily urinary tract infections (UTIs), respiratory infections and bacteraemia especially in immunocompromised individuals [1]. Next to Eschericia coli, *K. pneumoniae *is one of the most frequent causes of catheter-associated urinary tract infections (CAUTIs). The high incidence of CAUTIs has significant costs. Besides the economic aspect due to extended hospital admission days, the infection can spread to the kidneys and bloodstream causing systemic disease including bacteraemia [2-5].

The ability of bacteria to form biofilms on medical devices, e.g. catheters, is believed to play a major role in development of nosocomial infections including CAUTIs [2,5-7]. Biofilm formation, i.e. bacteria form an organized matrix-enclosed community adhering to the surface and each other, provides enhanced tolerance to antibiotics and the host immune system compared to growth as planktonic cells. Adhesion to the surface is the first essential step in biofilm formation; but adhesins may also play a significant role in later steps of biofilm development, e.g. by promoting cell-cell contact. Indeed, various fimbrial adhesins have been shown to play a role in biofilm formation in different bacterial species including *E. coli, Pseudomonas aeruginosa, Vibrio cholera *and *Vibrio parahaemolyticus *[8-12]. Most *K. pneumoniae *isolates express two types of fimbrial adhesins, type 1 and type 3 fimbriae [1]. Type 1 fimbriae are found in the majority of enterobacterial species; they mediate adhesion to mannose-containing structures and their expression is phase variable, i.e. mediated by an invertible DNA element (*fim *switch) [13]. Type 3 fimbriae are present in practically all *K. pneumoniae *isolates and mediate adhesion to several cell types in vitro [14,15]; nevertheless, the receptor for type 3 fimbriae has not yet been identified. Historically, type 3 fimbriae have not been associated with *E. coli *; however most recently two independent studies have for the first time reported type 3 fimbriae expression in *E. coli *strains encoded by conjugative plasmids [16,17].

We most recently investigated the role of type 1 and type 3 fimbriae in *K. pneumoniae *virulence in a UTI, a lung infection, and a gastrointestinal colonization model [18,19]. Type 1 fimbriae were found to be essential for the ability of *K. pneumoniae *to cause UTI, whereas type 3 fimbriae were not essential for virulence in the tested animal models [18,19]. In the present study we assessed the role of type 1 and type 3 fimbriae in *K. pneumoniae *biofilm formation.

## Methods

### Bacterial strains and growth conditions

*K. pneumoniae *C3091 is a clinical urinary tract infection isolate expressing type 1 and type 3 fimbriae [20,21]. The isogenic C3091 type 1 fimbriae mutant (C3091Δ*fim*), type 3 fimbriae mutant (C3091Δ*mrk*) and type 1 and type 3 fimbriae double mutant (C3091Δ*fim*Δ*mrk*) were previously described including verification of expected fimbrial expression [18,19]. Unless otherwise stated, bacteria were cultured at 37°C on solid or liquid Luria-Bertani (LB) medium. When appropriate, media were supplemented with the following concentrations of antibiotics: apramycin, 30 μg/ml; and chloramphenicol, 30 μg/ml.

### Construction of fluorescently-tagged strains

To observe biofilm formation by confocal laser scanning microscopy (CLSM), the C3091 wild type and its fimbriae-mutants were chromosomally-tagged by allelic exchange of the *lacIZ *genes with a cassette encoding fluorescent protein (yellow fluorescent protein (YFP) or cyan fluorescent protein (CFP)) under control of the modified lac promotor P_A1/04/03_, and chloramphenicol resistance flanked by regions homologous to regions up- and down-stream the *lacIZ *genes. The cassette was generated by a modification of a three-step PCR procedure as previously described [18,19,22]. All primers used are listed in Table 1. As the first step, the fluorescent protein and chloramphenicol encoding cassette was amplified from pAR116 (YFP) or pAR145 (CFP) using primer pair Ucas and Dcas [23]. Secondly, from C3091 chromosomal DNA a 403 bp region and a 460 bp region flanking the *lacIZ *genes, were amplified by PCR using primer pairs lacIUp-F, lacIUp-R and lacZDw-F, lacZDw-R, respectively. At their 5' ends, primer lacIUp-R and primer and lacZDw-F contained regions homologous to the primers Ucas and Dcas, respectively. In the third step, the flanking regions were added on each side of the fluorescent protein and chloramphenicol resistance cassette by mixing 100 ng of each fragment, followed by PCR amplification using primer pair lacIUp-F and lacZDw-R. The PCR product was purified and electroporated into C3091 wild type or its fimbriae mutants harboring the thermo-sensitive plasmid pKOBEGApra encoding the lambda Red recombinase. The fluorescently tagged strains were selected by growth on LB plates containing chloramphenicol at 37°C. Loss of the pKOBEGApra plasmid was verified by the inability of the tagged strains to grow on LB agar plates containing apramycin. Correct allelic exchange was verified by PCR analysis using primer pair UplacI and DwlacZ flanking the *lacIZ *region.

**Table 1:** Primers used in this study


**Primer name**	**Sequence 5' 3'**

UCas	CAAGAATTGCCGGCGGAT
DCas	GGTATTTCACACCGCATAGC
lacIUp-F	GCTGGAAGTAAAGGCTGTCG
lacIUp-R	ATCCGCCGGCAATTCTTGTCCGGATATGGCCTGCCTGTTTCT
lacZDw-F	GCTATGCGGTGTGAAATACCAGCTGTTGACTCCCCTGCGTGACC
lacZDw-R	TTTCCGTCGGGAAGATGTAG
UplacI	GCTCCACCGCCCTTTTG
DwlacZ	GTCGCCCCCACGGATTA
CAS168	GGGACAGATACGCGTTTGAT
CAS169	GGCCTAACTGAACGGTTTGA

### Flow chamber biofilms

Biofilms were grown in flow chambers with individual channel dimensions of 1 × 4 × 40 mm supplied with modified FAB medium [8]. The modified FAB medium was supplemented with glucose (100 mg l^-1^) as carbon source and isopropyl-thio-beta-galactoside (IPTG; 12 mg l^-1^) to ensure expression of fluorescent proteins from the P_A1/04/03 _promotor. The flow system was assembled and prepared as described previously [24]. A microscope cover slip of borosilicate (Knittel 24 × 50 mm st1; Knittel Gläser) was used as substratum. The flow chambers were inoculated by injecting approximately 2 × 10^6 ^cells, into each flow chamber with a small syringe. After inoculation, the flow chambers were left without flow for 1 h, and medium flow (0.2 or 0.8 mm s^-1^ corresponding to laminar flow and Re numbers of 0.3 and 1.3, respectively) was started using a Watson Marlow 205 S peristaltic pump and the system was incubated at 30°C.

### Microscopy and image acquisition

Biofilm formation was monitored by CLSM four, 24, 48, and 72 hours after inoculation. Microscopic observations and image acquisitions were performed with a Zeiss LSM 510 CLSM (Carl Zeiss, Jena, Germany) using a 40 ×/1.3 oil objective. The microscope was equipped with lasers, detectors and filter sets for detecting CFP and YFP fluorescence. Simulated three-dimensional images were generated using the IMARIS software package (Bitplane AG, Zürich, Switzerland).

### Quantification of biofilm formation and statistical analysis

For quantitative analysis of the biofilms, CLSM images were analysed by the computer program COMSTAT [25]. The total amount of biomass on the surface, the relative substratum coverage and the average thickness of the biofilm were calculated. Differences between the wild type and each mutant in the three parameters were compared by using a two-tailed independent t-test. P values below 0.05 were considered to be statistically significant.

### Fimbrial switch orientation assay

A modification of a previously described method was used to determine the orientation of the *fim*-switch in *K. pneumoniae *biofilms [18,26]. Biofilm samples were obtained by aspiration of the biofilm from individual flow cell channels by use of a syringe. All inoculum and biofilm samples were boiled for 5 min in PBS immediately after collection and then kept at -20°C until use. After thawing, the samples were boiled for 5 min, centrifuged at 12,000 g for 15 min and 2 μl of the supernatant used as template for PCR. Primers CAS168 and CAS169 (Table 1) were used to amplify an 817 bp region containing the *fim*-switch by use of the Expand High Fidelity PCR System (Roche). The PCR cycle conditions were as follows: 1 cycle of 94°C for 2 min; 30 cycles of 94°C for 15 s, 52°C for 1 min, 72°C for 1 min; one cycle of 72°C for 7 min. The PCR products were cut with *Hin*fI and separated on a 1.2% agarose gel. Due to asymmetric location of the *Hin*fI cleavage site inside the invertible element, different sized DNA fragments are obtained depending on the orientation of the phase switch.

## Results

### Role of fimbriae in *K. pneumoniae *biofilm formation by investigating monoculture biofilms

To investigate the role of type 1 and type 3 fimbriae in *K. pneumoniae *biofilm formation a well-defined isogenic type 1 fimbriae mutant (C3091Δ*fim*), a type 3 fimbriae mutant (C3091Δ*mrk*), and a type 1 and 3 fimbriae double mutant (C3091Δ*fim*Δ*mrk*) of the clinical UTI isolate C3091 were used. The wild type and its fimbriae mutants were found to have similar growth rates in the modified FAB medium used for biofilm experiments (results not shown).

**Figure 1:**
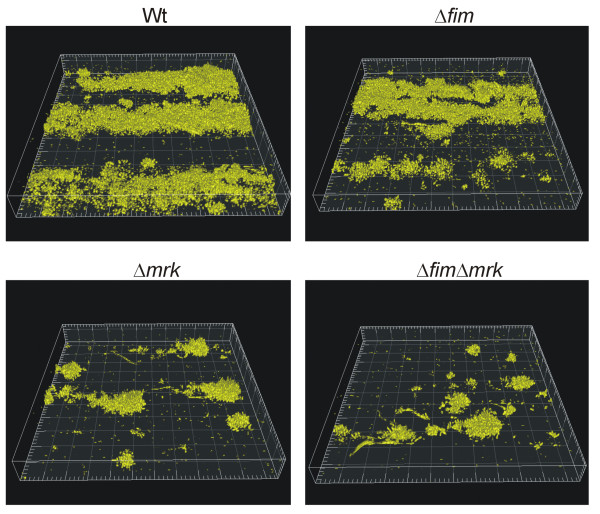
**One-day old biofilms of *K. pneumoniae *C3091 and its isogenic fimbriae mutants at flow 0.2 mm/s**. Biofilm formation was examined in three independent experiments with similar results. Box sides 230 μm × 230 μm.

Biofilm formation was observed four hours after inoculation of bacteria and after one, two, and three days. Four hours after inoculation of the flow-system, single cells of the wild type strain and its type 1 fimbriae mutant were observed adhering to the substratum whereas only very few cells of the type 3 fimbriae and the type 1 and 3 fimbriae double mutant were detected (results not shown). After 24 hours the wild type and the type 1 fimbriae mutant were found to form characteristic biofilms on the substratum observed as long extended colonies in the flow direction (Figure 1).

In contrast, the type 3 fimbriae mutant and the type 1 and 3 fimbriae double mutant only formed distinct microcolonies. Thus type 3 fimbriae, but not type 1 fimbriae, are important for attachment to the substratum as well as the initial stages of biofilm formation.

### Effect of flow on biofilm formation

**Figure 2:**
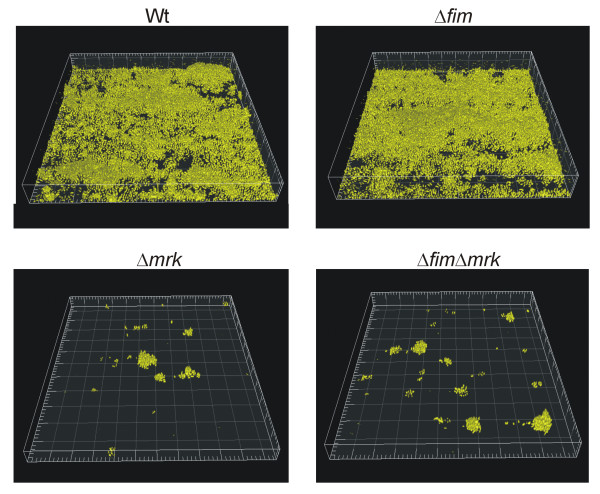
**One-day old biofilms of *K. pneumoniae *C3091 and its isogenic fimbriae mutants at flow 0.8 mm/s**. Biofilm formation was examined in three independent experiments with similar results. Box sides 230 μm × 230 μm.

To investigate the influence of shear forces on biofilm formation, a similar experiment was performed, except the media flow speed was raised from 0.2 mm/s to 0.8 mm/s. Under higher flow speed, the influence of type 3 fimbriae was even more pronounced (Figure 2). The two mutants unable to express type 3 fimbriae (C3091Δ*mrk *and C3091Δ*fim*Δ*mrk*) formed even fewer and smaller colonies. Also the biofilm formation of the wild type and the type 1 fimbriae mutant was influenced by the higher flow speed. Both cell types formed flat biofilms compared to biofilms under lower flow velocity, likely due to increased removal of loosely attached cells. However, the biofilms were significantly more pronounced and continuous and covered most of the surface compared to the biofilms of the type 3 fimbriae mutant and the type 1 and 3 fimbriae double mutant (Figure 2). In some areas the wild type and type 1 fimbriae mutant covered the whole surface as seen in Figure 2 and in other areas they formed the long extended colonies in the flow direction as observed under lower flow speed. This heterogeneity may be related to small differences in the flow cell micro-environment including lower flow stress due to presence of upstream biofim.

### Biofilm formation by wild type and mutants in competition

**Figure 3:**
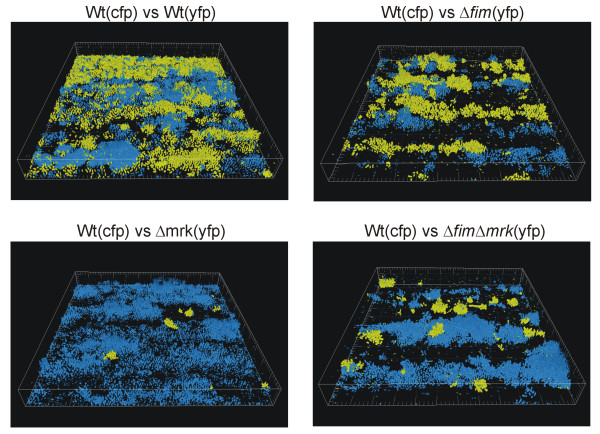
**Competition biofilm experiments with *K. pneumoniae *C3091 and its isogenic fimbriae mutants**. The pictures are of one day old biofilms. All biofilms were initiated with a 1:1 mixture of CFP-tagged and YFP-tagged bacteria. Biofilm formation was examined in three independent experiments with similar results. Box sides 230 μm × 230 μm.

To further characterize the influence of fimbriae on *K. pneumoniae *biofilm formation, flow cell experiments were performed with the different fimbriae mutants in direct competition with the wild type strain. For these experiments the wild type strain was chromosomally-tagged with cyan fluorescent protein (CFP). To verify that the YFP- and CFP-tagging did not have any influence on the biofilm formation, equal amounts of the YFP- and CFP-tagged wild type variants were inoculated in the same flow cell. As seen in Figure 3A, the biofilm formation of the YFP- and CFP-labelled wild types was similar. Furthermore, the results indicate that the *K. pneumoniae *biofilm develops primarily by clonal growth and not by recruitment of planktonic cells, as the biofilm was formed by large colonies of either YFP or CFP labelled cells. If the biofilm was developed by recruitment of planktonic cells, there would be a mix of YFP- and CFP-labelled cells in the colonies of the biofilm.

Competition experiments with the wild type and type 1 fimbriae mutant revealed that biofilm formation by the mutant strain were similar to the wild type (Figure 3B). As competition experiments are expected to reveal even minor differences in the ability to form biofilm, this verifies that type 1 fimbriae do not play a role in *K. pneumoniae *biofilm formation. In contrast the experiments with the C3091Δ*mrk *and C3091Δ*fim*Δ*mrk *mutants in competition with the wild type show a pronounced difference in biofilm formation (Figure 3C and 3D). In both cases the biofilm was formed by the wild type strain and only few small patches of the mutant strains were detected. Thus, the competition experiments confirmed that type 3 fimbriae are essential for *K. pneumoniae *biofilm formation.

### Quantitative analysis of biofilm formation by wild type and mutants

**Figure 4:**
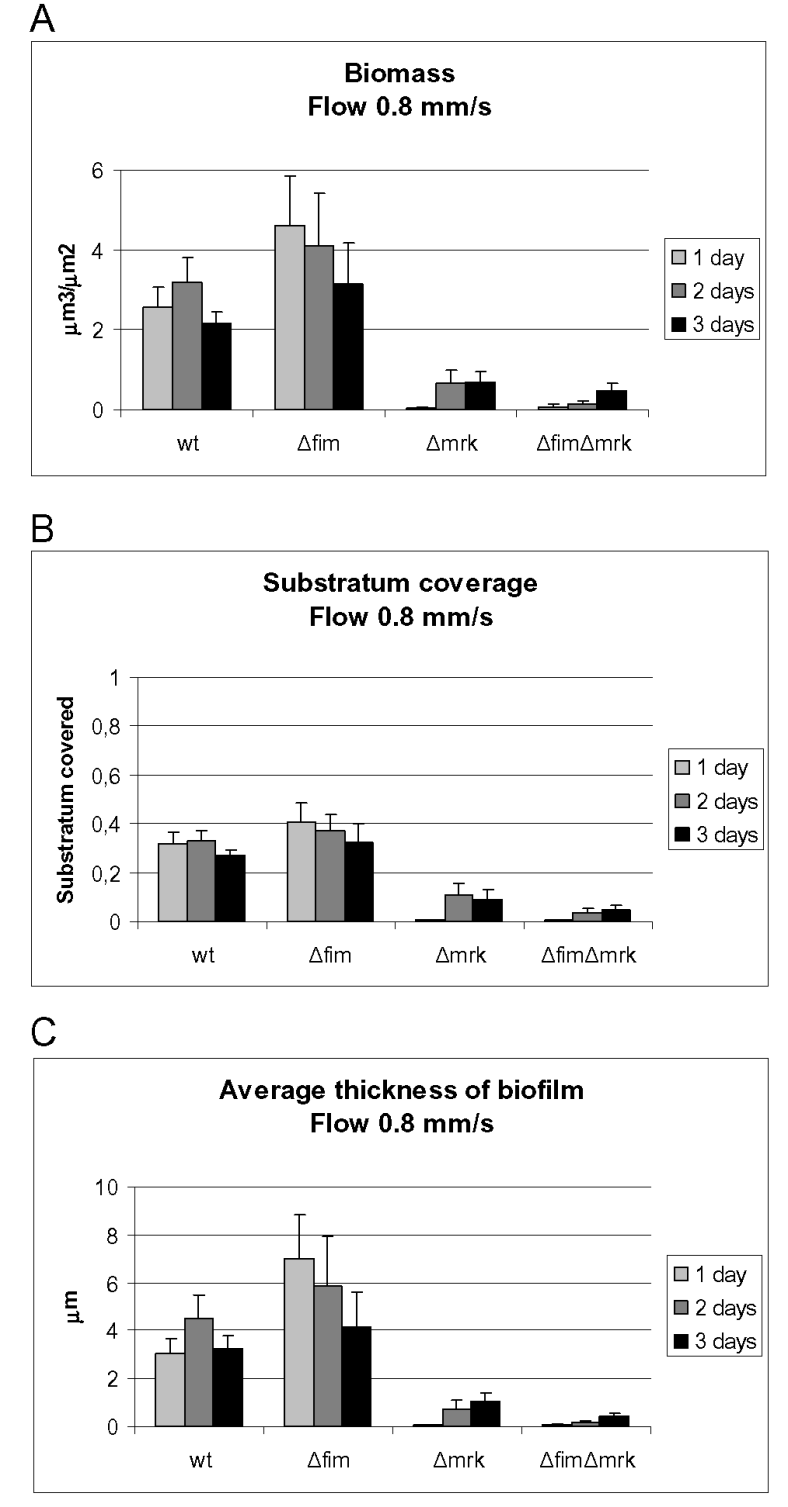
**Quantitative analysis of biofilm formation by *K. pneumoniae *C3091 and its isogenic fimbriae mutants at different time-points by use of the computer program COMSTAT**. A. Biomass. B. Substratum coverage (1 represents total coverage). C. Average thickness of biofilm. The mean and standard errors of the means are shown. Values were calculated from analysis of a minimum of seven images.

The computer program, COMSTAT [25], was used to quantitatively analyse the biofilm formed by the wild type and its fimbriae mutants. Three different parameters, biomass, substratum coverage, and average thickness, were calculated from CSLM images of biofilms formed one, two and three days after inoculation. The average biofilm biomass level and thickness did differ over the three day period indicating continuous biofilm development and adaptation during the time period. At all timepoints, the wild type and the type 1 fimbriae mutant formed significantly more biomass per surface area than the two mutants lacking the ability to form type 3 fimbriae (C3091Δ*mrk *and C3091Δ*fim*Δ*mrk*) (Figure 4A). No significant differences in biomass were detected between the wild type and the type 1 fimbriae mutant in the 1-3 days old biofilms. In contrast, a highly significant difference in biomass between the wild type and the type 3 fimbriae mutant (*P *< 0.01) and the type 1 and type 3 fimbriae double mutant was observed at all timepoints (*P *< 0.01).

Also the substratum coverage was significantly reduced for the type 3 fimbriae mutants in the 1-3 days old biofilms (Figure 4B). Both the type 3 fimbriae mutant and the type 1 and 3 fimbriae double mutant exhibited a much lower substratum coverage than the wild type (*P *< 0.01), whereas there was no significant difference between the wild type and the type 1 fimbriae mutant.

The average thickness of the 1-3 days old biofilms formed by the type 3 fimbriae mutant and the type 1 and 3 fimbriae mutant was also significantly lower than for the wild type (Figure 4C) (*P *< 0.01), while there was no significant difference between the wild type and the type 1 fimbriae mutant. Thus type 3 fimbriae do not only mediate cell-surface attachment to the substratum, but are also important for cell-cell adherence.

### Complementation by type 3 fimbriae restores biofilm formation of the mutant

**Figure 5:**
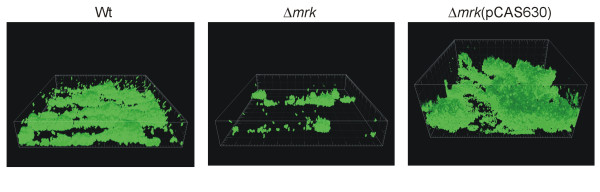
**Comparison of biofilm formation by the wild type, type 3 fimbriae mutant, and the type 3 fimbriae mutant transformed with pCAS630 containing the type 3 fimbriae gene cluster**. Biofilm formation was examined in three independent experiments with similar results. Box sides 230 μm × 230 μm.

To verify that the attenuated biofilm formation of the type 3 fimbriae mutants was due to abolishment of type 3 fimbriae expression and not polar effects of the mutation, the type 3 fimbriae mutant was transformed with pCAS630 containing the C3091 *mrk *gene cluster [19]. In contrast to the type 3 fimbriae mutant, the complemented mutant exhibited pronounced biofilm formation confirming the significant role of type 3 fimbriae in *K. pneumoniae *biofilm formation (Figure 5). In fact, the biofilm formation was even more prominent than for the wild type strain, likely due to enhanced type 3 fimbriae expression from the plasmid vector.

### Type 1 fimbriae expression is down-regulated in *K. pneumoniae *biofilms

**Figure 6:**
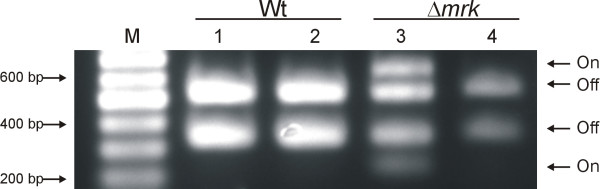
**Orientation of the *fim *phase switch in inoculum suspensions and biofilms of the wild type and type 3 fimbriae mutant (Δ*mrk*)**. Lane M contained molecular size markers. Lane 1, wild type Inoculum; lane 2, wild type biofilm; lane 3, Δ*mrk *inoculum; lane 4, Δ*mrk *biofilm. The lower band intensity in lane 4 is likely related to the low level of biofilm formed by the type 3 fimbriae mutant.

Expression of *K. pneumoniae *type 1 fimbriae is regulated by phase variation mediated by an invertible DNA element (*fim*-switch). To investigate the expression of type 1 fimbriae during biofilm formation, the orientation of the *fim*-switch in cells forming biofilm was compared with the orientation in the bacterial suspension used to inoculate the flow-cells. The switch orientation was investigated for the wild type as well as the type 3 fimbriae mutant. In the inoculum suspension of the wild type, only fragments corresponding to the switch orientation in the "off" orientation were detected (Figure 6). Also in the cells from wild type biofilm only the "off" orientation was detected.

Interestingly, in the inoculum suspension of the type 3 fimbriae mutant both the "on" and the "off" orientation was detected, indicating that abolishment of type 3 fimbriae expression leads to up-regulation of type 1 fimbriae expression. However, as for the wild type, only the "off" orientation was detected in type 3 fimbriae mutant biofilms. Thus, type 1 fimbriae expression was established to be down-regulated in *K. pneumoniae *biofilms even when the biofilm forming strains were unable to produce type 3 fimbriae.

## Discussion

The role of *K. pneumoniae *type 1 and type 3 fimbriae in vivo was recently investigated by our group [18,19]. Type 1 fimbriae were established to be an essential virulence factor in *K. pneumoniae *UTI whereas expression of type 3 fimbriae had no influence on pathogenicity in an UTI animal model. Furthermore, neither type 1 fimbriae nor type 3 fimbriae were found to influence the ability to colonize the intestinal tract or cause lung infection. The virulence studies were conducted by use of non-complicated mouse models and it could be speculated that the influence of fimbrial expression on virulence may be different in complicated infections, e.g. infections related to use of indwelling devices such as catheters [18,19]. It is well known that many pathogenic bacteria form biofilms on catheter surfaces, therefore we have in the present study characterized the influence of type 1 and type 3 fimbriae on *K. pneumoniae *biofilm formation.

The *K. pneumoniae *wild type strain was found to form characteristic biofilms in a continuous flow system. Single cells attached to the substratum followed by proliferation whereby micro-colonies were formed. Spread of the biofilm likely occurs by release of cells from the micro-colonies that subsequently attach to the substratum down-stream of the colony whereby characteristic long colonies are formed in the flow direction. In competition experiments, where the flow chambers were incubated with equal number of different colour-tagged isolates (YFP- and CFP-tagged), the colonies in the biofilms consisted of either yellow (YFP)- or blue (CFP) -tagged cells, rather than a mix of two colours (Figure 3). This indicates that recruitment of planktonic cells does not play a significant role in *K. pneumoniae *biofilm development. If recruitment of planktonic cells played a major role, the biofilm would be a mix of YFP- and CFP-tagged cells. Thus, our results reveal that development of *K. pneumoniae *biofilm occurs primarily by clonal growth.

The type 1 fimbriae mutant was found to be an as effective biofilm former as the wild type strain. Even when inoculated simultaneously with the wild type, the type 1 fimbriae mutant formed as much biofilm as the parent strain. Also quantitative analysis of the biofilms, using the computer program COMSTAT, revealed no significant difference in biomass, substratum coverage, and average thickness of the biofilm between the wild type and the type 1 fimbriae mutant. Equal amounts of substratum coverage indicate that type 1 fimbriae are not directly involved in cell-surface attachment. Furthermore, the similar biofilm biomass and thickness demonstrates that type 1 fimbriae are not involved in cell-cell adherence in the biofilm. Cover slips of borosilicate were used as substratum in our study and it can not be excluded that type 1 fimbriae may play a role in biofilm formation on other substratums.

It was most intriguing, that type 1 fimbriae was not involved in biofilm formation as type 1 fimbriae are an essential virulence factor in *K. pneumoniae *urinary tract infection [18,19] and seen to promote biofilm formation in *E. coli *[10,27]. Therefore, we investigated whether this lack of impact of type 1 fimbriae on biofilm formation was related to down-regulation of fimbrial expression. Type 1 fimbriae expression is regulated by the *fim*-switch containing the promoter for the major fimbrial subunit *fim*A [28]. The orientation of the *fim*-switch was investigated, in order to assess whether type 1 fimbriae were expressed during biofilm formation. Only the "off" orientation was detected from the C3091 wild type, demonstrating that type 1 fimbriae are down-regulated in biofilm forming cells. In contrast to the wild type, both the "on" and the "off" orientation was detectable in the inoculum suspension of the type 3 fimbriae mutants. Thus, abolishment of type 3 fimbriae expression was compensated by up-regulation of type 1 fimbriae, indicating cross-regulation of the two fimbrial gene clusters. We recently reported that the two fimbrial gene clusters are situated in close proximity on the *K. pneumoniae *chromosome, only interspaced by a 4.6 kb region which encodes putative regulatory genes [19]. Experiments to elucidate the putative cross-regulation of type 1 and type 3 fimbriae expression have been initiated in our group.

Although the type 3 fimbriae mutant expressed type 1 fimbriae in the suspension used to inoculate the flow chambers, only the "off" orientation was detected from the biofilm. Thus, even though the mutant was unable to express type 3 fimbriae, type 1 fimbrial expression was down-regulated, emphasizing that type 1 fimbriae do not play a significant role in biofilm formation. We previously demonstrated that type 1 fimbrial expression is up-regulated in wild type *K. pneumoniae *C3091 cells infecting the bladder (only "on" orientation detectable) but are down-regulated in C3091 cells colonizing the intestinal tract as well as when infecting the lungs (only "off" orientation detectable) [18]. That the *fim*-switch in different scenarios, including biofilms, are only detected in the "off" or the "on" orientation indicates either that specific environmental signals induce switching to either the "on" or "off" position or alternatively, that the specific environments provoke a strong selection for either fimbriated or non-fimbriated bacteria. In our experiments, if expression of type 1 fimbriae promoted biofilm formation, a selection of type 1 fimbriae producing variants, would be expected to occur during biofilm formation. This would especially be the case for the type 3 fimbriae mutant as cells expressing type 1 fimbriae were already present in bacterial suspension used to inoculate the flow chambers.

To our knowledge this is the first study which has investigated the influence of type 1 fimbriae on *K. pneumoniae *biofilm formation by use of well-defined isogenic mutants. It may be argued that the role of type 1 fimbriae in biofilm formation may be strain specific. However, supporting our findings, a previous study testing phenotypic expression of type 1 fimbriae in various *K. pneumoniae *isolates found that biofilm formation on plastic surfaces was not correlated with type 1 fimbrial expression [29].

In *E. coli *, a very close relative to *K. pneumoniae *, type 1 fimbriae have been shown to promote biofilm formation [10,27]. We are speculating that this intriguing difference may be related to the characteristic production of copious amounts of capsular material by *K. pneumoniae *strains. Indeed, it has been demonstrated that the presence of capsule is important for *K. pneumoniae *biofilm establishment and maturation [30]. Furthermore, capsule expression has been shown to inhibit type 1 fimbriae functionality [31,32]. Thus, it could be speculated, that up-regulation of capsule expression during biofilm formation inhibits type 1 fimbriae functionality, therefore type 1 fimbriae expression is down-regulated. Both the C3091 wild type and its fimbriae mutants are pronouncedly capsulated when grown on agar plates. We have initiated experiments to investigate the cross-regulation between capsule and fimbrial expression during *K. pneumoniae *biofilm formation.

In contrast to type 1 fimbriae, type 3 fimbriae were found to play an essential role in *K. pneumoniae *C3091 biofilm formation. The type 3 fimbriae mutants formed only sparse biofilms in the flow chambers and were essentially outcompeted when in direct competition with the wild type. The COMSTAT results for both the type 3 fimbriae mutant and type 1 and 3 fimbriae double mutant revealed much lower substratum coverage than the wild type. This indicates that type 3 fimbriae are most important for initial cell-surface attachment. Furthermore, the lower amount of biomass and average thickness of the biofilms for the type 3 fimbriae mutants compared to the wild type and type 1 fimbriae mutant indicates that type 3 fimbriae also mediates cell-cell adherence in the biofilm. Our results confirm previous studies demonstrating that type 3 fimbriae are important for *K. pneumoniae *biofilm formation [29,33]. Also in *E. coli *, the recently discovered ability to express type 3 fimbriae, mediated by conjugative plasmids, was found to profoundly enhance biofilm formation [16,17]. Thus, type 3 fimbriae expression seems to generally promote biofilm formation in different bacterial species.

We have previously established that type 1 fimbriae but not type 3 fimbriae are an essential virulence factor in *K. pneumoniae *urinary tract infections [18,19]. The present study demonstrates how the impact of a specific virulence factor may vary significantly in different infection scenarios and host environments. Thus, although type 3 fimbriae may not be significantly involved in development of uncomplicated UTIs, our results indicates that type 3 fimbriae may be a significant virulence factor in CAUTIs since they promote biofilm formation on inert surfaces. Understanding the mode of bacterial growth in vivo during infection is important in relation to future therapeutic measures.

## Conclusions

In conclusion, the present work shows that type 3 fimbriae, but not type 1 fimbriae, mediate biofilm formation in *K. pneumoniae *C3091. As type 3 fimbriae promote adhesion to abiotic surfaces and biofilm formation in *K. pneumoniae *and other species, as shown here and by other studies [16,17,29,33], type 3 fimbriae may generally play a significant role in development of catheter related infections such as CAUTIs. In this respect, the occurrences of conjugative plasmids encoding type 3 fimbriae in other species are worrisome.

As the vast majority of *K. pneumoniae *isolates are able to express both type 1 and type 3 fimbriae [1], the use of epidemiological studies to elucidate the role of fimbriae in catheter associated *K. pneumoniae *infections is difficult. Thus further studies using catheterized in vivo infection models, are needed to further characterize the role of fimbriae in catheter related infections.

## Authors' contributions

CSC, KAK and CST participated in the design of the study. CSC and CST constructed the fluorescently labeled strains and performed the fimbrial switch assays. CSC and KBB performed the biofilm experiments. All authors participated in data analysis and drafted the manuscript. All authors read and approved the final manuscript.

## References

[B1] Podschun R Ullmann U *Klebsiella spp*. as nosocomial pathogens: epidemiology, taxonomy, typing methods, and pathogenicity factors Clin Microbiol Rev 1998 11 589 603 9767057 10.1128/cmr.11.4.589 PMC88898

[B2] Maki DG Tambyah PA Engineering out the risk for infection with urinary catheters Emerg Infect Dis 2001 7 342 347 10.3201/eid0702.010240 11294737 PMC2631699

[B3] Ronald A The etiology of urinary tract infection: traditional and emerging pathogens Am J Med 2002 113 Suppl 1A 14S 19S 10.1016/S0002-9343(02)01055-0 12113867

[B4] Stamm WE Catheter-associated urinary tract infections: epidemiology, pathogenesis, and prevention Am J Med 1991 91 65S 71S 10.1016/0002-9343(91)90345-X 1928194

[B5] Warren JW Catheter-associated urinary tract infections Int J Antimicrob Agents 2001 17 299 303 10.1016/S0924-8579(00)00359-9 11295412

[B6] Donlan RM Costerton JW Biofilms: survival mechanisms of clinically relevant microorganisms Clin Microbiol Rev 2002 15 167 193 10.1128/CMR.15.2.167-193.2002 11932229 PMC118068

[B7] Stewart PS Costerton JW Antibiotic resistance of bacteria in biofilms Lancet 2001 358 135 138 10.1016/S0140-6736(01)05321-1 11463434

[B8] O'Toole GA Kolter R Flagellar and twitching motility are necessary for *Pseudomonas aeruginosa *biofilm development Mol Microbiol 1998 30 295 304 10.1046/j.1365-2958.1998.01062.x 9791175

[B9] Paranjpye RN Strom MS A *Vibrio vulnificus *type IV pilin contributes to biofilm formation, adherence to epithelial cells, and virulence Infect Immun 2005 73 1411 1422 10.1128/IAI.73.3.1411-1422.2005 15731039 PMC1064924

[B10] Pratt LA Kolte R Genetic analysis of *Escherichia coli *biofilm formation: roles of flagella, motility, chemotaxis and type I pili Mo Microbiol 1998 30 285 293 10.1046/j.1365-2958.1998.01061.x 9791174

[B11] Shime-Hattori A Iida T Arita M Park KS Kodama T Honda T Two type IV pili of *Vibrio parahaemolyticus *play different roles in biofilm formation FEMS Microbiol Lett 2006 264 89 97 10.1111/j.1574-6968.2006.00438.x 17020553

[B12] Watnick PL Fullner KJ Kolter R A role for the mannose-sensitive hemagglutinin in biofilm formation by *Vibrio cholerae *El Tor J Bacteriol 1999 181 3606 3609 10348878 10.1128/jb.181.11.3606-3609.1999 PMC93833

[B13] Klemm P Schembri P Bacterial adhesins: function and structure Int J Med Microbiol 2000 290 27 35 11043979 10.1016/S1438-4221(00)80102-2

[B14] Hornick DB Allen BL Horn MA Clegg S Adherence to respiratory epithelia by recombinant *Escherichia coli *expressing *Klebsiella pneumoniae *type 3 fimbrial gene products Infect Immun 1992 60 1577 1588 1312518 10.1128/iai.60.4.1577-1588.1992 PMC257033

[B15] Tarkkanen AM Allen BL Westerlund B Holthofer H Kuusela P Risteli L Clegg S Korhonen TK Type V collagen as the target for type-3 fimbriae, enterobacterial adherence organelles Mol Microbiol 1990 4 1353 1361 10.1111/j.1365-2958.1990.tb00714.x 1980713

[B16] Burmølle M Bahl MI Jensen LB Sørensen SJ Hansen LH Type 3 fimbriae, encoded by the conjugative plasmid pOLA52, enhance biofilm formation and transfer frequencies in *Enterobacteriaceae *strains Microbiology 2008 154 187 195 10.1099/mic.0.2007/010454-0 18174137

[B17] Ong CL Ulett GC Mabbett AN Beatson SA Webb RI Monaghan W Nimmo GR Looke DF McEwan AG Schembri MA Identification of type 3 fimbriae in uropathogenic *Escherichia coli *reveals a role in biofilm formation J Bacteriol 2008 190 1054 1063 10.1128/JB.01523-07 18055599 PMC2223576

[B18] Struve C Bojer M Krogfelt KA Characterization of *Klebsiella pneumoniae *type 1 fimbriae by detection of phase variation during colonization and infection and impact on virulence Infect Immun 2008 76 4055 4065 10.1128/IAI.00494-08 18559432 PMC2519443

[B19] Struve C Bojer M Krogfelt KA Identification of a conserved chromosomal region encoding *Klebsiella pneumoniae *type 1 and type 3 fimbriae and assessment of the role of fimbriae in pathogenicity Infect Immun 2009 77 6592 6601 10.1128/IAI.00585-09 PMC2772557 19703972

[B20] Oelschlaeger TA Tall BD Invasion of cultured human epithelial cells by *Klebsiella pneumoniae *isolated from the urinary tract Infect Immun 1997 65 2950 2958 9199471 10.1128/iai.65.7.2950-2958.1997 PMC175413

[B21] Struve C Forestier C Krogfelt KA Application of a novel multi-screening signature-tagged mutagenesis assay for identification of *Klebsiella pneumoniae *genes essential in colonization and infection Microbiology 2003 149 167 176 10.1099/mic.0.25833-0 12576590

[B22] Derbise A Lesic B Dacheux D Ghigo JM Carniel E A rapid and simple method for inactivating chromosomal genes in *Yersinia* FEMS Immunol Med Microbiol 2003 38 113 116 10.1016/S0928-8244(03)00181-0 13129645

[B23] Reisner A Molin S Zecher EL Recombinogenic engineering of conjugative plasmids with fluorescent marker cassettes FEMS Microbiol Ecology 2002 42 251 259 10.1111/j.1574-6941.2002.tb01015.x 19709285

[B24] Christensen BB Sternberg C Andersen JB Palmer RJ Nielsen AT Givskov M Molin S Molecular tools for study of biofilm physiology Methods Enzymol 1999 310 20 42 full_text 10547780 10.1016/s0076-6879(99)10004-1

[B25] Heydorn A Nielsen AT Hentzer M Sternberg C Givskov M Ersboll MK Molin S Quantification of biofilm structures by the novel computer program COMSTAT Microbiology 2000 146 2395 2407 11021916 10.1099/00221287-146-10-2395

[B26] Struve C Krogfelt KA In vivo detection of *Escherichia coli *type 1 fimbrial expression and phase variation during experimental urinary tract infection Microbiology 1999 145 2683 2690 10537190 10.1099/00221287-145-10-2683

[B27] Schembri MA Klemm P Biofilm formation in a hydrodynamic environment by novel FimH variants and ramifications for virulence Infect Immun 2001 69 1322 1328 10.1128/IAI.69.3.1322-1328.2001 11179294 PMC98023

[B28] Abraham JM Freitag CS Clements JR Eisenstein BI An invertible element of DNA controls phase variation of type 1 fimbriae of *Escherichia coli* Proc Natl Acad Sci USA 1985 82 5724 5727 10.1073/pnas.82.17.5724 2863818 PMC390624

[B29] Di Martino P Cafferini N Joly B Darfeuille-Michaud A *Klebsiella pneumoniae *type 3 pili facilitate adherence and biofilm formation on abiotic surfaces Res Microbiol 2003 154 9 16 10.1016/S0923-2508(02)00004-9 12576153

[B30] Balestrino D Ghigo JM Charbonnel N Haagensen JA Forestier C The characterization of functions involved in the establishment and maturation of *Klebsiella pneumoniae *in vitro biofilm reveals dual roles for surface exopolysaccharides Environ Microbiol 2008 10 685 701 10.1111/j.1462-2920.2007.01491.x 18237304

[B31] Matatov R Goldhar J Skutelsky E Sechter I Perry R Podschun R Sahly H Thankavel K Abraham SN Ofek I Inability of encapsulated *Klebsiella pneumoniae *to assemble functional type 1 fimbriae on their surface FEMS Microbiol Lett 1999 179 123 130 10.1111/j.1574-6968.1999.tb08717.x 10481096

[B32] Schembri MA Dalsgaard D Klemm P Capsule shields the function of short bacterial adhesins J Bacteriol 2004 186 1249 1257 10.1128/JB.186.5.1249-1257.2004 14973035 PMC344426

[B33] Langstraat J Bohse M Clegg S Type 3 fimbrial shaft (MrkA) of *Klebsiella pneumoniae*, but not the fimbrial adhesin (MrkD), facilitates biofilm formation Infect Immun 2001 69 5805 5812 10.1128/IAI.69.9.5805-5812.2001 11500458 PMC98698

